# Sprachverstehen im Störschall – Überlegungen zur ökologisch validen Bewertung der Kommunikationsfähigkeit mit Cochleaimplantat

**DOI:** 10.1007/s00106-022-01234-1

**Published:** 2022-10-27

**Authors:** Matthias Hey, Alexander Mewes, Thomas Hocke

**Affiliations:** 1 Klinik für Hals-Nasen-Ohren-Heilkunde, Kopf- und Halschirurgie; Audiologie, UKSH, Campus Kiel, Arnold-Heller-Straße 14, 24105 Kiel, Deutschland; 2 Cochlear, Hannover, Deutschland

**Keywords:** Hörhilfen, Cochlea Implantat, Sprachaudiometrie, Sprachverständlichkeitsschwelle, Reliabilität und Validität, Hearing aids, Cochlear implant, Speech audiometry, Speech reception threshold, Reliability and validity

## Abstract

**Hintergrund:**

Heutzutage zeigen Patienten mit einem Cochleaimplantat (CI) meistens ein gutes bis sehr gutes Verstehen in Ruhe, berichten jedoch immer wieder über Probleme bei der Kommunikation in alltäglichen Nebengeräuschen. Um die akustische Komplexität dieser realen Hörsituationen bei der apparativen Versorgung von schwerhörigen Patienten zu berücksichtigen, besteht ein Bedarf an ökologisch validen Messungen des Sprachverstehens. Der damit verbundene methodische Mehraufwand muss mit personellen und räumlichen klinischen Ressourcen in Übereinstimmung gebracht werden. In der vorliegenden Studie werden mögliche Vereinfachungen einer komplexen Messanordnung untersucht.

**Methode:**

In die Studie wurden 20 Erwachsene aus der Langzeitnachsorge nach CI-Versorgung mit postlingualem Beginn der Hörstörung eingeschlossen. Die Komplexität der untersuchten Hörsituation wurde durch Veränderung der Räumlichkeit der Störschallquellen und durch den zeitlichen Charakter des Störschalls beeinflusst. Die verschiedenen Messanordnungen wurden mithilfe von unilateral gemessenen Sprachverständlichkeitsschwellen („speech reception thresholds“, SRT) verglichen, wobei verschiedene CI-Prozessoren und Einstellungen zum Einsatz kamen. Als Referenz dienten 10 normalhörende Probanden.

**Ergebnisse:**

In einer komplexen Hörsituation mit 4 Lautsprechern und fluktuierendem Störschall zeigten sich in den SRT Unterschiede zwischen CI-Trägern und der Kontrollgruppe von bis zu 8 dB. Für die CI-Träger korrelierten diese SRT mit der Situation mit frontalem Sprachsignal und fluktuierendem Störsignal von der Seite mit R^2^ = 0,69. Für Konditionen mit stationärem Störsignal fanden sich R^2^ < 0,2.

**Schlussfolgerungen:**

Bei der Räumlichkeit und dem zeitlichen Charakter von Störquellen gibt es keine universelle Lösung für alle audiometrischen Fragestellungen. Im hier beschriebenen Kontext ist eine Vereinfachung der komplexen räumlichen audiometrischen Anordnung mit Beibehaltung des fluktuierenden Störsignals möglich.

## Abstract (en)

**Background:**

Nowadays, cochlear implant (CI) patients mostly show good to very good speech comprehension in quiet, but there are known problems with communication in everyday noisy situations. There is thus a need for ecologically valid measurements of speech comprehension in real-life listening situations for hearing-impaired patients. The additional methodological effort must be balanced with clinical human and spatial resources. This study investigates possible simplifications of a complex measurement setup.

**Methods:**

The study included 20 adults from long-term follow-up after CI fitting with postlingual onset of hearing impairment. The complexity of the investigated listening situations was influenced by changing the spatiality of the noise sources and the temporal characteristics of the noise. To compare different measurement setups, speech reception thresholds (SRT) were measured unilaterally with different CI processors and settings. Ten normal-hearing subjects served as reference.

**Results:**

In a complex listening situation with four loudspeakers, differences in SRT from CI subjects to the control group of up to 8 dB were found. For CI subjects, this SRT correlated with the situation with frontal speech signal and fluctuating interference signal from the side with R^2^ = 0.69. For conditions with stationary interfering signals, R^2^ values <0.2 were found.

**Conclusion:**

There is no universal solution for all audiometric questions with respect to the spatiality and temporal characteristics of noise sources. In the investigated context, simplification of the complex spatial audiometric setting while using fluctuating competing signals was possible.

## Sprachverstehen in realitätsnahen Hörsituationen

Im Rahmen der Versorgung von schwerhörigen Patienten mit apparativen Hörsystemen besteht insbesondere in der Nachsorge ein Bedarf an Messungen des Sprachverstehens, welche verschiedene Aspekte der alltäglichen Nutzung abdecken. Dies bedeutet, dass im Rahmen der audiometrischen Untersuchungen in der Klinik/Praxis ein realitätsnahes Abbild der typischen täglichen Kommunikationssituationen geschaffen wird. Das weiterführende Ziel der so gewonnenen Ergebnisse kann die adäquate Bewertung der Defizite von Hörpathologien sein. Wichtige Merkmale von realen und alltagsrelevanten Hörsituationen sind u. a. fließende Sprachpräsentation, multimodale Stimuli, realistische Sprachpegel, räumliche Trennung von Signal- und Störschallquellen, Nachhall und das Auftreten von direktem und reflektiertem Schall, konkurrierender Störschall mit akustischen Eigenschaften unterschiedlicher Sprecher [9, 26, 42].

Bei Patienten mit hochgradigen Schwerhörigkeiten, die mit einem Hörgerät kein ausreichendes Sprachverstehen mehr erzielen können, besteht die Möglichkeit der (Wieder‑)Erlangung des Hörvermögens durch die Versorgung mit einem Cochleaimplantat (CI). Nach dieser operativen Therapie muss regelhaft eine Kontrolle des erzielbaren Sprachverstehens erfolgen. Dies dient der Charakterisierung des alltagsrelevanten Hörhandicaps, um im Weiteren das Verstehen zu optimieren. Es besteht weitgehend Einigkeit darüber, dass sowohl das Sprachverstehen in Ruhe als auch im Rauschen geeignete Surrogatparameter zur Beschreibung der Kommunikationsfähigkeit im Alltag sind [10, 29].

Das hierfür zur Verfügung stehende Methodeninventar besteht aus etablierten Untersuchungsmethoden wie z. B. dem Freiburger Sprachtest in Ruhe [15, 23, 24]. Bei seiner Verwendung kann die Beurteilung der Ergebnisse im Kontext anderer funktionsdiagnostischer Messungen durch mehr als ein halbes Jahrhundert an klinischer Erfahrung gestützt werden [15, 30]. Typische Anwendungsbereiche des Freiburger Einsilbertests sind die Diagnostik von Hörstörungen [33], die Indikation zu apparativer Versorgung [10], deren begleitende Erfolgskontrolle [17, 23, 24] und die Verwendung als Zielparameter pharmakologischer Studien [36]. Wie im Rahmen der CI-Versorgung gezeigt, können Einsilbertests zur Identifikation therapiebeeinflussender Faktoren [4, 20] und zur individuellen Prognose [14, 23] des postoperativen Sprachverstehens herangezogen werden. Diese Tests werden unter weitgehend standardisierten Bedingungen, d. h. Messung im Freifeld bei frontalem Schalleinfall bzw. unter Kopfhörern, durchgeführt [3, 7].

Ergänzende Untersuchungen erscheinen aufgrund ihrer besonderen Eignung zur Beantwortung spezieller Fragestellungen im konkurrierenden Störschall geeignet [8, 40, 42]. Hier wurde das Methodeninventar durch alternative Lautsprecheranordnung [7, 31, 40, 44] und Störsignale [8, 12, 21] erweitert. Bei einer zunächst ausschließlich wissenschaftlichen Fragestellung ist ein mit erhöhtem methodischem Aufwand realisierter Versuchsaufbau begründbar. Im Rahmen der Patientenversorgung sind es nicht nur limitierte räumliche und personelle Kapazitäten in der Funktionsdiagnostik, welche die Anzahl und den Umfang der durchzuführenden Tests begrenzen. Auch die Bewältigung eines standardisierten Messprotokolls kann für einige Patienten eine Belastung darstellen [24].

Die Weiterentwicklung der CI-Prozessortechnik hat zu einem verbesserten Sprachverstehen im Störschall geführt [11, 18, 31]. Allerdings zeigt sich bei der klinischen Umsetzung, dass CI-Träger von einer stärker individualisierten Einstellung ihrer Systeme über das etablierte Maß hinaus [24] profitieren können [19, 35, 39]. Hieraus ergeben sich mit erhöhtem zeitlichem Aufwand für den Patienten verbundene neue Testkonditionen. Um den damit verbundenen erhöhten Arbeitsaufwand für die Klinik zu begegnen, ist z. B. die von Rader et al. [39] vorgeschlagene Anpassmethode zur individuellen Wahl der Parameter des Sprachprozessors ein sinnvoller Ansatz. Sie lässt durch aktive und eigenständige Mitarbeit des Patienten [35] eine Entlastung des Klinikpersonals zu. Gleiches gilt auch für unlängst beschriebene einfache sprachaudiometrische Testverfahren auf der Basis mobiler Endgeräte [27]. Für komplexere Hörsituationen ist dies derzeit nur begrenzt möglich. So sind bei der Untersuchung von CI-Störschallunterdrückungsalgorithmen mit Richtwirkung die Untersuchung der Hörverbesserung durch das erforderliche Setup mit mehr als 2 Lautsprechern [11, 18, 31] nicht überall möglich. Die Mikrofoncharakteristik ForwardFocus (Fa. Cochlear Limited, Sydney, Australien) [18] ist ein für die sog. Cocktailpartysituation [37] entwickelter Algorithmus. Ein verbessertes Sprachverstehen wurde hier insbesondere für anspruchsvolle realitätsnahe Hörsituationen beschrieben [22]. Für diese Hörsituationen wurde ein audiometrisches Setting für die Messung des Sprachverstehens im fluktuierenden Störschall erstellt, welches eine hohe ökologische Validität [26, 42] aufweist. Dieses ist geprägt durch den Einsatz von fließender Sprache durch den Einsatz eines Satztests. Außerdem tragen räumlich verteilte Signalquellen mit frontaler Sprachpräsentation und konkurrierendem Störschall aus mehreren nichtkohärenten Quellen der hinteren Hemisphäre zur Realitätsnähe bei. Beim fluktuierenden Störsignal handelt es sich um ICRA-Rauschen (International Collegium of Rehabilitative Audiology) [12], welches männliche Sprecher aus mehreren Richtungen simuliert. Die Ergebnisse der CI-Patienten werden in Relation zu Normalhörenden gesetzt [40]. Dies erfolgte durch die Verwendung einer geeigneten Referenzmetrik [16, 18].

In der vorliegenden Arbeit wird untersucht, ob und inwieweit diese komplexe Hörsituation zur Messung des Verstehens im Störschall vereinfacht werden darf. Diese Vereinfachung wird in 2 Dimensionen betrachtet. Hierfür werden zum einen unterschiedliche zeitliche Charakteristika des Störschalls gewählt, stationäres sprachsimulierendes Oldenburger Rauschen im Vergleich zum fluktuierenden ICRA-Rauschen. Zum anderen werden vereinfachte räumliche Anordnungen der Störschallquelle(n) untersucht, Lautsprecherkonstellation mit 2 oder nur einer Signalquelle im Vergleich zur Referenzkonfiguration mit 4 Lautsprechern. Die Ergebnisse für Sprachverständlichkeitsschwellen (SRT), gemessen mit dem Oldenburger Satztest, werden in verschiedenen audiometrischen Settings verglichen und hinsichtlich ihrer Äquivalenz bewertet. Zusätzlich wird durch die Verwendung zweier Sprachprozessorgenerationen untersucht, inwieweit die technische Entwicklung selbiger zu einem verbesserten Sprachverstehen in diesen komplexen Hörsituationen beitragen kann.

## Methode

### Patienten

Eine Gruppe von 20 postlingual ertaubten CI-Patienten nahm an dieser Studie teil. Diese wurde vorab durch die örtliche Ethikkommission (D 06/18) genehmigt. Alle Untersuchungen wurden in Übereinstimmung mit den ethischen Standards der institutionellen und nationalen Forschungskommission sowie mit der Erklärung von Helsinki von 1964 und ihren späteren Änderungen oder vergleichbaren ethischen Standards durchgeführt.

Die Einschlusskriterien für die erwachsenen Studienteilnehmer waren ein postlinguales Einsetzen der Schwerhörigkeit und die Versorgung mit einem CI vom Typ CI24RE oder CI5xx (Fa. Cochlear Limited, Sydney, Australien) bei vollständiger Insertion des Elektrodenträgers in die Scala tympani. Die Teilnehmer mussten bei einer Eingangsuntersuchung im Oldenburger Satztest in Ruhe (65 dB_SPL_) ein Sprachverstehen von mindestens 80 % erzielen. Eine beidseitige Implantation war kein Ausschlusskriterium, aber es wurde im Rahmen dieser Studie jeweils nur ein Ohr pro Patient untersucht. Bei 17 Patienten waren alle 22 Elektroden aktiviert, in 3 Fällen (#8; 10; 16) waren 21 Elektroden aktiv.

Das Durchschnittsalter der Teilnehmer betrug 53 Jahre (Minimum: 31 Jahre, Maximum: 76 Jahre). Die Teilnehmer hatten eine mittlere CI-Erfahrung von 8,3 Jahren (mindestens 6,0 Jahre, höchstens 15,4 Jahre). Diese biografischen Details der Teilnehmer wurden bereits publiziert [16].

Für die Referenzierung des Verstehens der CI-Patienten auf die Vergleichsdaten Normalhörender wurden zusätzlich 10 normalhörende erwachsene Probanden rekrutiert und bei allen Untersuchungen monaural untersucht. Das Gegenohr wurde passiv vertäubt mittels Ohrstöpsel und Kapselkopfhörer. Bei jedem dieser Teilnehmer wurde eine tonaudiometrische Untersuchung durchgeführt, um sicherzustellen, dass sie ein normales Hörvermögen gemäß DIN ISO 8253:3 [25] im Frequenzbereich von 250–8000 Hz aufwiesen.

### Testverfahren

In dieser Studie wurden wiederholte Messungen an den gleichen Probanden durchgeführt. Die Tests wurden in 3 Testsitzungen (randomisierte Reihenfolge) im Abstand von 2–3 Wochen durchgeführt, um eine Gewöhnung an unterschiedliche Sprachprozessoren und Signalverarbeitungsalgorithmen im Alltag zu ermöglichen.

Alle Tests wurden in einer akustisch geschirmten Hörkabine (ISO 8253:227) durchgeführt. Die Lautsprecher befanden sich 1,3 m vom Patienten entfernt. Es wurden folgende Lautsprecherkonfigurationen genutzt:

- S0N0 – Sprache und Störschall frontal
- S0N90 – Sprache frontal und Störschall 90° ipsilateral zum untersuchten CI
- S0N90, 180, 270 – Sprache frontal und Störschall aus 90°, 180° und 270°

Sätze im Störgeräusch wurden mit einer computergestützten Implementierung des Oldenburger Satztests (Equinox-Audiometer; Fa. Interacoustics, Middelfart, Dänemark, und evidENT3-Software; Fa. Merz Medizintechnik, Reutlingen, Deutschland) dargeboten. Die Oldenburger Sätze [8] wurden bei konstantem Störschallpegel von 65 dB_SPL_ dargeboten. Als Störschall kam zum einen das stationäre sprachsimulierende Oldenburger Rauschen und zum anderen das fluktuierende ICRA-Rauschen [12] zum Einsatz. Für Letzteres wurde Track-Nr. 5 der ICRA-CD genutzt, welche die spektrale und temporale Charakteristik eines einzelnen männlichen Sprechers aufweist. Bei der Störschallpräsentation aus der hinteren Hemisphäre (S0N90, 180, 270) wird das Rauschen aus den unterschiedlichen Richtungen nichtkohärent präsentiert. Die SRT wurde mithilfe eines adaptiven Verfahrens [5] gemessen und ist definiert als das Signal-Rausch-Verhältnis („signal-to-noise ratio“, SNR), das ein 50%ig korrektes Wortverstehen ergibt. Alle CI-Träger waren das adaptive Testverfahren gewöhnt, da sie im Rahmen der klinischen Routine bereits 5 oder mehr Mal getestet worden waren. Um eine ausreichende Minimierung des prozeduralen Lerneffekts zu gewährleisten, wurde zu Beginn jeder Testsitzung ein zusätzliches Training durchgeführt (30 Sätze bei 65 dB_SPL_). Die Messung des Sprachverstehens erfolgte stets monaural. Kontralaterale CI wurde für den Messvorgang entfernt bzw. ein kontralaterales Restgehör passiv vertäubt mittels Ohrstöpsel und Kapselkopfhörer.

Bei jedem Untersuchungstermin in der Klinik wurden die CI-Sprachprozessoren technisch überprüft. Bei Bedarf wurden Systemkomponenten ausgetauscht.

Alle Patienten verwendeten die ACE-Kodierungsstrategie („advanced combination encoder“) mit individuell angepasster Stimulationsrate und Anzahl der Maxima. Die individuellen Map-Parameter (T- und C‑Level) der CI-Sprachprozessoren wurden über den gesamten Zeitraum der Studie unverändert genutzt. Jedoch wurden die Algorithmen der akustischen Signalvorverarbeitung entsprechend dem Studienprotokoll verändert. Es kamen dabei jeweils die Signalverarbeitungen zum Einsatz, die vom Szenenklassifizierer [31] im Störschall aktiviert werden: CP910 mit der Mikrofoncharakteristik Beam (CP9Beam; Beam, Cochlear Ltd.) und CP1000 ebenso mit Beam (CP10Beam) [41]. Diese wurden mit der manuellen Einstellung im Prozessor CP1000 unter Einsatz von ForwardFocus (CP10FF) verglichen. Außerdem wurden immer die Signalvorverarbeitungen ADRO („automatic dynamic range optimization“; Cochlear Ltd.), ASC („automatic sensitivity control“) und SNR-NR (Störgeräuschunterdrückung; Cochlear Ltd.) aktiviert [31, 34]. Nach einer 2‑ bis 3‑wöchigen Eingewöhnungszeit an den jeweiligen Sprachprozessor wurden die audiometrischen Tests durchgeführt.

### Datenauswertung

Zur Visualisierung des alltagsrelevanten Hördefizits wurden die SRT der CI-Träger relativ zum Verstehen von Normalhörenden in der gleichen Situation aufgetragen [16, 18]. Zum Vergleich der verschiedenen Messkonditionen wurden paarweise intraindividuelle Vergleichsanalysen mit Bonferroni-Korrektur durchgeführt. Es wurde ein Signifikanzniveau von 0,05 zur Bestimmung der Signifikanz für 2‑seitige Analysen verwendet.

Die Daten wurden als Boxplotgrafiken dargestellt. Dargestellt sind der Median (durchgezogene Mittellinie), die 25- und 75-Perzentil-Intervalle (Boxlimits) sowie die 5‑ und 95%-Perzentile (Whisker). Der Mittelwert ist als ein quadratisches Symbol eingezeichnet.

## Ergebnisse

**Figure 1:**
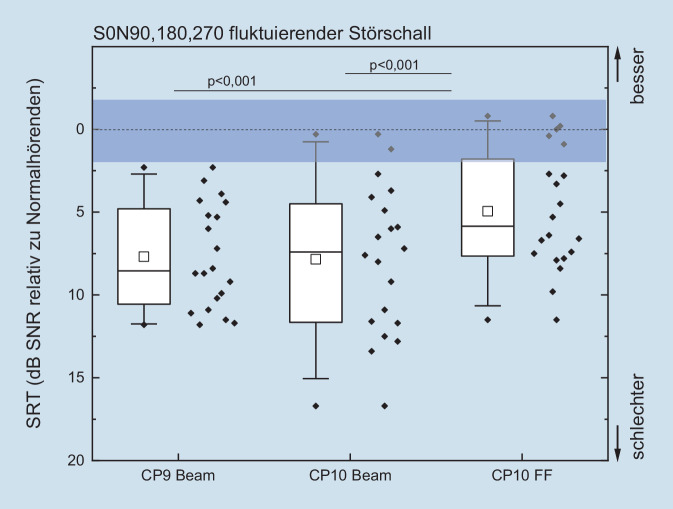

Alle Studienteilnehmer haben die Untersuchungen im Störschall erfolgreich absolviert. In Abb. 1 ist das Sprachverstehen in der komplexesten Hörsituation dieser Studie dargestellt. Es ist die SRT im fluktuierenden Störschall für die Lausprecherkonstellation S0N90, 180, 270 in Abhängigkeit vom Sprachprozessor und dessen Einstellung relativ zum monauralen Sprachverstehen Normalhörender aufgetragen. Dabei wurde eine signifikante Verbesserung um ≈ 3 dB_SNR_ für den Sprachprozessor CP10 beim Wechsel von Beam auf ForwardFocus bei frontaler Sprachpräsentation und fluktuierendem Störschall aus der hinteren Hemisphäre festgestellt. Bei Einsatz des Sprachprozessors CP10 erreichten einzelne CI-Patienten den monauralen Referenzbereich von Normalhörenden. Zum Vergleich mit den Ergebnissen sind in Tab. 1 die Mittelwerte und Standardabweichungen der SRT des Referenzkollektivs aufgeführt.

**Table 1:** 

Messkondition	Mittelwert der SRT (dB SNR)	Standardabweichung der SRT (dB SNR)
S0N0 stationärer Störschall	−8,2	1,8
S0N0 fluktuierender Störschall	−26,0	2,6
S0N90 stationärer Störschall	−8,1	2,0
S0N90 fluktuierender Störschall	−24,9	3,5
S0N90, 180, 270 stationärer Störschall	−2,2	0,9
S0N90, 180, 270 fluktuierender Störschall	−18,1	1,9

*SNR *Signal-Rausch-Verhältnis („signal-to-noise ratio“),* SRT* Sprachverständlichkeitsschwelle („speech reception threshold“)

**Figure 2:**
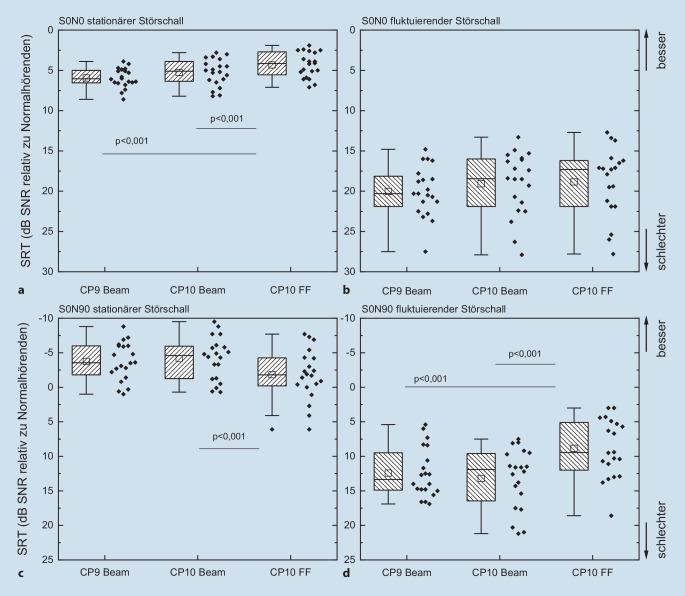

Die 3 untersuchten Sprachprozessorkonfigurationen wurden weiterführend mit reduzierter räumlicher Lautsprecherkonstellation (S0N0 und S0N90) im stationären und fluktuierenden Störschall untersucht (Abb. 2), wobei die Sprache stets frontal dargeboten wurde. Dabei zeigt sich ein deutlich besseres Verstehen im stationären Störschall im Vergleich zum fluktuierenden Störschall. So erzielte die Mehrzahl der CI-Patienten im Vergleich zu Normalhörenden ein besseres Verstehen in S0N90 im stationären Störschall.

**Figure 3:**
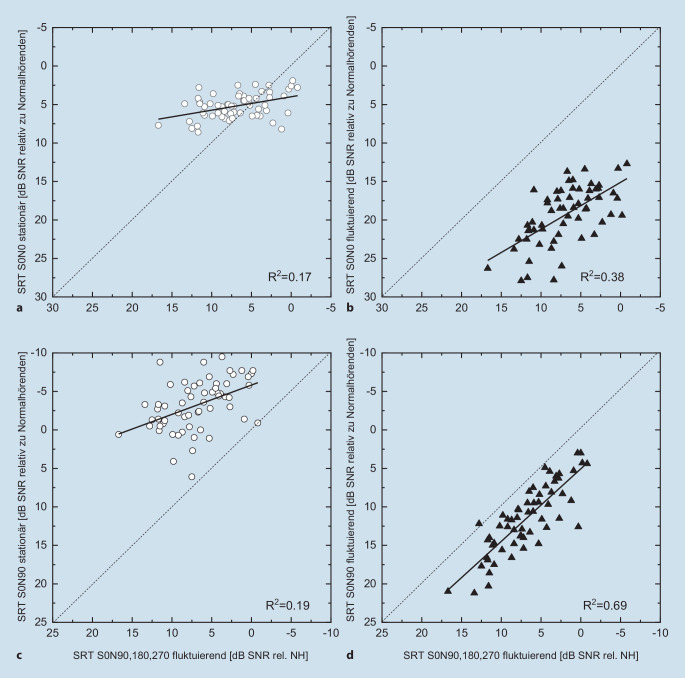

In Abb. 3 wurden die SRT in der ökologisch validen Situation S0N90, 180, 270 im fluktuierenden Störschall in Abhängigkeit vom Verstehen in den anderen Untersuchungs-Setups (Abb. 2) aufgetragen. Die Korrelation zum Verstehen im stationären Störschall war mit R^2^ = 0,17 (S0N0) bzw. 0,19 (S0N90) nur gering. Deutlich stärker ausgeprägt war die Korrelation zum Verstehen im fluktuierenden Störschall mit R^2^ = 0,38 (S0N0) und zeigte den größten Wert für die Lautsprecherkonfiguration S0N90 von R^2^ = 0,69. In diesem Fall verlief die Regressionsgerade weitgehend parallel zur Winkelhalbierenden.

## Diskussion

### Methodik der Sprachaudiometrie

Ausgangspunkt der Studie war ein komplexer audiometrischer Aufbau zur Untersuchung des Verstehens im fluktuierenden und räumlich separierten Störschall (S0N90, 180, 270). Die Verwendung von mehreren Lautsprechern zur Erfassung des Gewinns durch Signalvorverarbeitung in CI-Systemen ist eine etablierte Methode [6, 45]. Zusätzlich kann durch die Auswahl eines geeigneten Störschalls die ökologische Validität für eine spezielle Alltagssituation erhöht werden. So können alltägliche störschallbehaftete Hörsituationen wie z. B. bei einer Familienfeier oder bei einem Besuch in einem Restaurant in ihrer Charakteristik nachgebildet werden [26, 37, 42]. Es wurde untersucht, ob und inwieweit dieser Messaufbau vereinfacht werden darf. Als Basis diente dabei ein 4‑Lautsprecher-Setting mit fluktuierenden Störsignalen aus mehreren nichtkohärenten Quellen der hinteren Hemisphäre. Dieses Untersuchungs-Setup ist nicht Teil des Standardrepertoires der klinischen Sprachaudiometrie. Eine Vereinfachung ist über 2 Wege möglich: Veränderung der Anordnung und der Anzahl der Lautsprecher sowie die Auswahl eines geeigneten kompetitiven Signals. Das räumliche Setting wurde über 2 Lautsprecher (S0N90) bis hin zu frontalem Schalleinfall für Sprache und Störschall (S0N0) aus einem Lautsprecher vereinfacht. Außerdem wurde das fluktuierende ICRA5-Störsignal, welches die spektralen und temporalen Eigenschaften eines einzelnen Sprechers aufweist, mit dem klinisch etablierten stationären sprachsimulierenden Störsignal (Rauschen des Oldenburger Satztests) verglichen.

Die Abb. 3a und c zeigen, dass sich das als für eine Cocktailpartysituation repräsentative Störsignal nicht durch ein stationäres Rauschen ersetzen lässt. Die SRT zeigen sowohl für die Signalquellenkonfiguration S0N0 als auch für S0N90 nur eine geringe Korrelation mit dem Referenzsetting (S0N90, 180, 270). Wird jedoch das kompetitive Signal beibehalten und nur die Lautsprecheranordnung auf S0N90 vereinfacht, zeigt sich eine hohe Korrelation mit dem Referenzsetting. Die Reduzierung des methodischen Aufwands zur Bestimmung des Sprachverstehens in einer ökologisch validen Hörsituation ist also in gewissen Grenzen möglich. Die Korrelation der Ergebnisse in Abb. 3 in den einzelnen Settings zeigt, dass die Lautsprecheranordnung von 4 auf 2 reduziert werden kann. Hingegen ist ein Wechsel von einem fluktuierenden auf ein stationäres Störsignal nicht angeraten.

Die Verwendung fluktuierender Störsignale ist geeignet, alltagsrelevante Hörsituationen in der Audiometrie abzubilden [40, 44]. Die „derzeit verwendeten sprachaudiometrischen Verfahren berücksichtigen zwar weitgehend standardisierte Bedingungen“, jedoch limitiert der vergleichsweise niedrige Komplexitätsgrad ihre Fähigkeit, Alltagssituationen („ökologische Validität“) abzubilden [32]. Trotz der schon sehr früh beschriebenen Relevanz komplexerer Situationen [37] für das Hören im Alltag ist die Verwendung komplexerer Störsignale in der klinischen Routine eher wenig verbreitet. Man kann annehmen, dass die Verwendung fluktuierender Störsignale einer durchaus anzustrebenden Standardisierung in der Sprachaudiometrie entgegenwirkt, da sich derzeit kein Signal als universell einsetzbar erwiesen hat. Bisher sind je nach wissenschaftlicher Fragestellung ein ganzes Spektrum verschiedener Signale [12, 40, 44] zur Anwendung gekommen. Nur der Einsatz komplexer kompetitiver Signale erlaubt die Erfassung von Zielgrößen zur Beschreibung bzw. Verbesserung des Verstehens in anspruchsvollen Hörsituationen [38, 44]. Umso erfreulicher sind Vorschläge verschiedener Arbeitsgruppen, welche eine Standardisierung fördern können [12, 13].

### Referenzierung auf Normalhörende

Die Auseinandersetzung mit komplexen Hörsituationen ist geprägt von spezieller und technisch anspruchsvoller Methodik. Hinzu kommt, dass die Ergebnisse durch andere Arbeitsgruppen nur schwer zu interpretieren sind. Abhilfe kann hier die Referenzierung auf Normalhörende bieten, wie sie in dieser und anderen Arbeiten [16, 18, 40, 44] eingesetzt wurde.

In Abb. 2 sind die SRT der CI-Träger in Relation zu normalhörenden Probanden dargestellt. Für die CI-Träger finden sich im Setting S0N90 mit stationärem Störgeräusch sehr niedrige SRT im Vergleich zur normalhörenden Kontrollgruppe. Hierbei handelt es sich um ein künstlich geschaffenes, vermeintliches Abbild einer alltäglichen Hörsituation. Ziel ist es hier, die Verbesserung für die Patienten durch geeignete Signalvorverarbeitung audiometrisch abzubilden. Der Einsatz von Beamformern führt hier sehr eindrücklich zu einer Verbesserung der SRT in dieser speziellen Situation, und dies sogar in einer Weise, dass die überwiegende Mehrheit der CI-Träger ein besseres Verstehen als die Normalhörenden zeigt. Grund hierfür ist die ideale Eignung der Beamformer für dieses Untersuchungs-Setup. Diese Argumentation stellt nicht den Nutzen der Beamformer infrage, aber vor dem Hintergrund der bekannten Probleme der CI-Patienten im Störschall [1, 43] erzeugt dieses Ergebnis erhebliche Zweifel an der ökologischen Validität eben dieser Messanordnung, S0N90 mit stationärem Störschall. Auf diesen Widerspruch wurde unlängst durch Badajoz-Davila und Buchholz [2] hingewiesen: „… standard speech-in-noise tests overestimate the performance of cochlear implant recipients in the real world. To address this limitation, future assessments need to improve the realism over current tests by considering the realism of both, the speech and the noise materials.“ Insofern sind Bedenken bzgl. des Einsatzes von stationärem Störschall insbesondere vor dem Hintergrund der Diskussion um die Verwendung komplexerer Störsignale zur Beschreibung von ökologisch validen Hörsituationen [32] berechtigt. Jedoch darf sich die Diskussion hinsichtlich einer möglichst hohen die ökologischen Validität nicht nur auf die Komplexität einer (Test‑)Situation bzw. der in ihr verwendeten Signale beschränken. Auch und vor allem hängt sie mit der Hörumgebung der verschiedenen mit einem CI versorgten Menschen zusammen. Das kann bei einem Patienten durch ein stationäres Motorengeräusch bestimmt sein, während andere eher die Situation in Ruhe als ihre Lebenswirklichkeit begreifen. Oberhoffner et al. weisen in ihrer Arbeit u. a. auch auf die sich mit dem Lebensalter veränderten Hörgewohnheiten und -umgebungen hin. Außerdem ist nach Ansicht der Autoren noch nicht abschließend geklärt, inwieweit die sinnfreien Sätze des Oldenburger Satztests eine realistische Abbildung der Lebenswirklichkeit der Patienten darstellen. Insbesondere dieser Sachverhalt muss Gegenstand weiterführender Untersuchungen sein.

### Ziele der Audiometrie

Man kann im Rahmen von audiometrischer Diagnostik unterscheiden zwischen

- audiometrischen Verfahren zur Diagnostik,
- therapiebegleitenden Verlaufskontrollen und
- audiometrischen Verfahren zu weiterführenden Fragestellungen.

Audiometrische Verfahren zur Diagnostik eines Hör‑/Verstehensdefizits und zur Beschreibung des Ausmaßes und der Lokalisation einer Schädigung müssen nicht zwingend ökologisch valide sein. Sie sollen eine Therapieentscheidung unterstützen.

Therapiebegleitenden Verlaufskontrollen [10, 28] dienen dem Ziel des Monitorings der zeitlichen Entwicklung bei fortschreitender Rehabilitation. Dies zielt auf die Dokumentation der zeitlichen Entwicklung, aber auch auf die frühe Erkennung eines möglichen Verfehlens von Therapiezielen ab.

Audiometrische Verfahren zur Adressierung weiterführender wissenschaftlich/klinischer Fragestellungen können der Optimierung der Kommunikationsfähigkeit der betroffenen Patienten in ihrer Lebenswirklichkeit dienen. Sie sollten sich nahe an der akustischen Alltagsrealität dieser Patienten orientieren und damit eine möglichst hohe ökologische Validität aufweisen. Es ist gerade die Alltagsrealität, die eine ständige Veränderung dieser Methoden impliziert. Sowohl der Alltag der Patienten als auch die erweiterten technischen und medizinischen Optionen bestimmten die Methodik.

Die aktuellen Diskussionen zu ökologischer Validität finden ihre Wurzeln schon im Kontext der Anfänge der deutschensprachigen Audiometrie: „Die Entwicklung von Physik und Technik hat in den letzten Jahren und Jahrzehnten dem Arzt eine Fülle neuer Möglichkeiten der Diagnostik und Therapie in die Hand gegeben. […] Auf akustischem Gebiet ist die Audiometrie heute zu einem feinen, ja allerfeinsten diagnostischen Instrument ausgebaut. Schon die richtige Handhabung der Reintonaudiometrie erfordert Kenntnisse und viel praktische Erfahrung. Etwas komplizierter liegen die Dinge noch bei der Sprachaudiometrie, die einerseits den Vorteil hat, dass mit ihr die Gesamtheit des Hörens komplexer Lautgebilde gemessen werden kann, der aber andererseits die Schwierigkeiten einer Messmethode anhaften, in deren Ergebnis eine große Zahl von Faktoren eingeht. Trotz des größeren Aufwandes an Apparaturen und Sachkenntnis ist diese Methode aber heute unentbehrlich sowohl für die Begutachtung der Hörfähigkeit schlechthin oder der Gehörsänderung durch therapeutische Eingriffe als auch ganz besonders für die Anpassung von Hörgeräten“. Dieses über 60 Jahre alte Zitat von Zöllner [46] hat nichts an seiner Aktualität eingebüßt. Es unterstreicht die Notwendigkeit des steten Ringens um bestmögliche Diagnostik und Therapie. Im Angesicht weiterentwickelter Verfahren in der HNO-Heilkunde muss die Sprachaudiometrie immer wieder überdacht werden.

## Fazit für die Praxis

- In der therapiebegleitenden Diagnostik von Hörstörungen gibt es keine universelle Lösung für alle audiometrischen Fragestellungen.
- Zusätzlich zu den etablierten Standardverfahren wie dem Freiburger Sprachtest und den im deutschsprachigen Raum genutzten Satztesten im stationären Rauschen sind neue, an die speziellen Fragestellungen/Therapieverfahren angepasste Tests notwendig.
- Ein komplexes audiometrisches Setting zum Sprachverstehen, bestehend aus 4 Lautsprechern mit fluktuierendem Maskierer aus der hinteren Hemisphäre, kann auf 2 Lautsprecher unter Beibehaltung des fluktuierenden Maskierers reduziert werden und führt zu vergleichbaren audiometrischen Ergebnissen im hier untersuchten Kontext.
